# Update Overview of the Role of Angiopoietins in Lung Cancer

**DOI:** 10.3390/medicina57111191

**Published:** 2021-11-01

**Authors:** Dimitris Tsakogiannis, Asimina Nikolakopoulou, Flora Zagouri, Grigorios Stratakos, Konstantinos Syrigos, Eleni Zografos, Nikolaos Koulouris, Garyfalia Bletsa

**Affiliations:** 1 Research Center, Hellenic Anticancer Institute, 10680 Athens, Greece; dtsakogiannis@gmail.com; 2 Intensive Care Unit, 1st Department of Respiratory Medicine, Medical School, National and Kapodistrian University of Athens, 11527 Athens, Greece; mn_nikol@yahoo.com (A.N.); grstrat@hotmail.com (G.S.); koulnik@med.uoa.gr (N.K.); 3 Department of Clinical Therapeutics, School of Medicine, Alexandra Hospital, National and Kapodistrian University of Athens, 11528 Athens, Greece; florazagouri@yahoo.co.uk (F.Z.); el_zogra@hotmail.com (E.Z.); 4 Third Department of Internal Medicine, Sotiria Hospital, National and Kapodistrian University of Athens, 11527 Athens, Greece; ksyrigos@med.uoa.gr

**Keywords:** angiopoietins, angiogenesis, lung cancer, non-small-cell lung cancer, Ang-1, Ang-2, Ang-3, Ang-4

## Abstract

Angiogenesis is a biological process that involves the formation of new blood vessels from the existing vasculature, and it plays a fundamental role in the development and progression of several types of cancer, including lung cancer. The angiopoietin/Tie2 ligand/receptor system orchestrates vascular integrity. In particular, Angiopoietin-1 activates the endothelial cell (EC)-specific receptor tyrosine kinase, Tie2, which is essential for preserving endothelial quiescence. On the other hand, Angiopoietin-2 acts as an inhibitor of the Angiopoietin-1/Tie2 signaling pathways, thus facilitating the destabilization of quiescent endothelium in cases of inflammation and cancer. Clinical studies have proven that high levels of Angiopoietin-2 indicate the development of non-small-cell lung carcinomas (NSCLC), while high levels of Angiopoietin-2 are strongly related to tumor angiogenesis, lymphangiogenesis, metastasis, and poor prognosis. Interestingly, the association of Angiopoietin-2 levels with the type of surgical approach makes Angiopoietin-2 a valuable factor in selecting the most suitable therapeutic strategy for lung cancer patients. The role of the Angiopoietin-1 and Angiopoietin-4 levels in NSCLC development requires further investigation. The present review focuses on the clinical impact of the Angiopoietin-1, Angiopoietin-2, and Angiopoietin-4 levels in patients diagnosed with NSCLC, emphasizing the interaction between them, and how they affect the development, progression, and metastasis of lung disease. Finally, it estimates the role of angiopoietins levels in the effective therapy of lung cancer patients.

## 1. Introduction

Lung cancer is a substantially invasive and promptly metastasizing type of cancer, with the five-year survival rate of the disease expected to reach approximately 16% [1]. Epidemiologic findings have revealed that, in 2018, more than 2 million new lung cancer incidences were recorded, accounting for more than 1.5 million cancer-associated deaths globally [2]. In particular, it has been estimated that lung cancer is the most frequent type of cancer among men, followed by prostate and colorectal cancer, and it is the leading cause of cancer-associated deaths in males. With regard to the female population, lung cancer constitutes the third most common type of cancer, rating after breast and colorectal cancer, whereas it is the second most prevalent cause of cancer-related deaths, after breast cancer [2,3]. Although tobacco smoking is the highest risk factor for the growth and mortality motif of lung cancer, the disease can also develop in nonsmokers, with the origin of tumorigenesis in these cases linked to environmental and genetic factors [4,5,6].

Lung cancer is regarded as a heterogeneous type of malignancy that can affect any element of the respiratory system, thus leading to the development of a wide range of symptoms that are mainly affected by the anatomic location of the malignancy. With regard to the morphology of tumor cells, lung cancer is classified into two different histological groups with different patterns of growth, metastasis, and treatment: small-cell lung cancer (SCLC) and non-small-cell lung carcinomas (NSCLC). Interestingly, non-small lung cancer is diagnosed in 85% of lung cancer incidences, while the most common subtypes of NSCLC are adenocarcinoma (LUAD), and lung squamous cell carcinoma (LUSC) [7,8]. Nowadays, several treatments have been developed targeting several factors, including epidermal growth factor receptor (EGFR),vascular epidermal growth factor receptor (VEGFR), phosphatidylinositol 3-kinase (PI3Ks), anaplastic lymphoma kinase (ALK), and v-Raf murine sarcoma viral oncogene homolog B (BRAF) [9,10]. Although the established treatment approaches have substantially improved patient survival, there is growing interest in identifying novel biomarkers with prognostic and predictive value in order to enable patient stratification, as well as biomarkers that may be used as therapeutic targets alone, or in combination with existing treatments. 

Nowadays, several studies have implied that angiogenenesis plays a pivotal role in the development, spread, and metastatic dissemination in several types of cancers, including lung cancer [11,12,13,14]. Angiogenesis is a physiological procedure that promotes the recruitment of new blood vessels and it is induced by an imbalance between angiogenic and antiangiogenic factors. The most widely known angiogenic factors are vascular endothelial growth factor (VEGF), VEGF receptors (VEGFRs), and angiopoietins [15]. VEGF promotes the proliferation, migration, and spread of endothelial cells [14,16]. The expression of VEGF and VEGFRs are upregulated in solid tumors and they significantly contribute to cancer development and dissemination [17,18]. The overexpression of VEGF and VEGFRs has been observed in both NSCLC and SCLC [19]. The angiopoietin (Ang) family is composed of three angiopoietins, namely, Ang-1, Ang-2, and Ang-4 (the mouse ortholog is named Ang-3), which are ligands of theendothelial cell (EC)-specific receptor tyrosine kinase, Tie-2. Receptor tyrosine kinases are produced in the endothelial cells (ECs) of blood and lymphatic vessels and regulate the growth and function of the cardiovascular and lymphatic systems. The angiopoietin ligand/Tie receptor pathway is essential for the remodeling of blood and lymphatic vessels during embryonic and postnatal growth, as well as for the preservation of the mature vasculature [20,21]. Angiopoietin-1 (Ang-1) stimulates vessel stabilization and maturation, while its role in the development of cancer remains rather vague [22,23]. Angiopoietin-2 (Ang-2) is a proangiogenic factor that is expressed at low levels in normal tissues, and is extensively expressed in tumor endothelial cells. It has been estimated that predominantly Ang-2, in association with VEGF and other proangiogenic factors, triggers tumor angiogenesis [24,25]. Angiopoietin-4 (Ang-4) and Angiopoietin-3 (Ang-3) seem to act as agonists of the Tie-2 receptor, while their role in tumor angiogenesis and invasion is unclear [18,25]. 

Lung cancer is a form of cancer that often lacks signs and symptoms until it becomes advanced, leading to a low survival rate. Numerous efforts have been made to identify new markers of prognosis, diagnosis, and therapy. Angiogenesis is a complex process, and several preclinical and clinical studies have been focused on the VEGF/VEGFR signaling pathway. It remains a challenge to comprehend the exact role of each angiogenic factor in lung cancer initiation, progression, and treatment. The angiopoietin family, and mainly Ang-2, is another predominant angiogenic factor implicated in lung cancer. The aim of the present review is to examine the role of angiopoietins in all aspects regarding lung cancer. In particular, it describes the role of angiopoietins in the development of lung cancer, and examines the value of angiopoietins as prognostic/diagnostic tools, their significance after surgery, and their potential as a future therapeutic target.

## 2. Angiopoietins

Angiopoietins consist of a small group of secreted glycoproteins that are implicated in the growth, modification, and recovery of the blood vessels. Angiopoietin family members (Ang-1, Ang-2, Ang3, and Ang-4) contain a small amino-terminal domain that is essential for the clustering of proteins (termed the “superclustering region”), a large central coiled-coil pattern, and a C-terminal fibrinogen domain. Finally, a short linker bridges the central coiled-coil motif and the fibrinogen domain [26,27,28,29]. The fibrinogen domain is involved in the binding of angiopoietins to Tie2, while the coiled-coil motif and the N-terminal domain are required for the oligomerization of angiopoietins, which is essential for Tie-2 activation [26,27,28,29]. Ang-1 is regarded as a strong Tie-2 agonist. In addition, Ang-4 and the mouse orthologue, Ang-3, are Tie-2 agonists, while their role in angiogenesis remains rather unclear [30]. Ang-2 has been regarded as an antagonist of Tie-2 that competitively restrains Ang-1 binding. However, more recent studies imply that Ang-2 has a dual impact on angiogenesis, since it acts either as a weak Tie-2 agonist or as a Tie-2 antagonist [31,32,33]. 

The oligomerized form of Ang-1 (tetrameric form or higher-order multimeric structures) links Tie-2 molecules at the endothelial cell–cell joints, thus leading to the transactivation of Tie-2 [34,35]. Subsequently, Tie-2 promotes the selective stimulation of Akt that, in turn, contributes to vascular quiescence through the activation of theAkt–Foxo1 and Akt–eNOS pathways. In particular, Akt phosphorylates and inhibits the transcriptional factor forkhead box protein, O1 (FOXO1), while it promotes the phosphorylation of eNos [36,37]. The Akt–Foxo1 signaling pathway is implicated in the growth and metabolism of endothelial cells, while theAkt–eNos pathway is essential for vascular maturation [36,37]. On the other hand, Ang-2 is found in lower-order multimeric structures (mainly as dimmers, trimers, or tetramers). Notably, the oligomerization status of angiopoietins influences the agonist capacity of the protein [38,39,40]. Specifically, Ang-2, as a lower-order multimeric structure, is considered a weak Tie-2 agonist, since Tie-2 activation is initiated by high-order multimeric angiopoietins, such as Ang-1 [31,32,33,34,41]. The agonist function of Ang-2 is essential for the development of the lymphatic vasculature, whereas its antagonist capacity facilitates the inhibition of the Ang-1/Tie-2 signaling pathways in cases of inflammation and cancer [31,34,37].

## 3. Ang-1 

Although Ang-1 has a crucial impact on angiogenesis, serum Ang-1 levels are not usually measured in human cancers, and its role in lung cancer growth remains rather unclear. The overexpression of Ang-1 exerts considerable influence on blood vessel stabilization, tumor angiogenesis, and metastasis in mice [42,43]. Ιn patients diagnosed with NSCLC, contradictory outcomes concerning theserum levels of Ang-1 have emerged. Initially, it was suggestedthatAng-1 expression has no clinical significance with regard to the prognosis and survival of patients with lung cancer [44]. In contrast, Park et al. [23] first revealed that patients diagnosed with NSCLC exhibit lower serum concentrations of Ang-1 compared to the control group, while patients with higher levels of Ang-1 have better disease and relapse-free survival. These observations led to the assumption that the augmented serum concentrations of Ang-1enable the prediction of patient survival after surgery, and the disease recurrence in patients in the early stages of NSCLC [23]. On the other hand, a relatively recent study found higher serum levels of Ang-1 in NSCLC patients compared to the control cohort, while no further association between the concentration of Ang-1 and the treatment’s outcome was identified [45]. This discrepancy in the results could be associated with the different genetic backgrounds of the studied populations. Hence, further analyses are required in order to test this hypothesis, as well as to assess whether genetic or epigenetic factors may modify the expression of Ang-1 in patients with lung cancer and, consequently, to clarify whether there is a genetic implication of the Ang-1 gene in the lung cancer risk among different ethnic groups [46,47].

With regard to lung cancer tissue, the clinical significance of the Ang-1 levels is controversial as well [23,48,49,50]. Even though the concentration of Ang-1 in lung cancer tissues seems to be downregulated, it is uncertain whether Ang-1 can be considered a valuable biomarker [23,45,48,49,50]. This contradiction lies mainly in the fact that Ang-1 exhibits excessively variable expression, even in samples collected from the same tissue, while it should be considered that a small tissue section is not indicative of the entire tissue [23,48,49,50]. This variability of Ang-1 expression in lung cancer tissues might influence the serum Ang-1 levels, thus leading to contradictory findings among different studies. Therefore, Ang-1 should be examined in combination with additional angiogenic factors, including Ang-2 and VEGF, in order to draw a more accurate conclusion considering lung cancer progress and recurrence [23,45]. 

## 4. Ang-2

The expression of Ang-2 in the presence of VEGF is the biological hallmark of blood vessel destabilization, which eventually results in cancer growth and, subsequently, tumor progression. Nowadays, it is well-established that the serum levels of Ang-2 are significantly associated with the onset and progression of NSCLC [23,24,25,51,52,53,54]. Although high serum levels of Ang-2 are detected in patients diagnosed with NSCLC, considerable interest has developed concerning the prognostic value of Ang-2. In particular, Park et al. [54] first reported that patients with low serum levels of Ang-2 exhibited a better overall survival compared to patients with higher levels of Ang-2. However, various research analyses followed without drawing definite conclusions [44,55,56,57,58]. Notably, a recent meta-analysis reinforced the initial observation of Park et al. [54] supporting that Ang-2 exhibits strong prognostic value in both NSCLC and SCLC incidences [52]. This association seems to be stronger in SCLC cases compared to NSCLC cases. Considering that SCLC is a more aggressive type of cancer, it was concluded that Ang-2 might influence the severity and invasion of the disease [52]. The strong association of the serum levels with the severity of lung disease was also recorded among the different stages of lung cancer, with the highest serum Ang-2 levels recorded in stage IV. In addition, serum Ang-2 levels have been associated with tumor dissemination, since high levels of Ang-2 were also found in patients with lymph node metastasis [52].

Even though the research studies have focused on Ang-2 serum level measurements in lung cancer cases, comparable results were recorded when circulating Ang-2 mRNA levels were determined in the blood of lung cancer patients [59,60]. In particular, significantly increased levels of Ang-2 mRNA were recorded in cases with NSCLC, while it was observed that patients with elevated Ang-2 mRNA levels exhibit limited overall survival when compared to patients with lower levels of the corresponding mRNA. This association was found to be stronger in patients diagnosed with stage IV NSCLC, supporting that high Ang-2 mRNA levels come along with the severity of the disease [59,60]. Consequently, both the serum Ang-2 levels and the Ang-2 mRNA levels serve as strong prognostic tools of NSCLC. 

With regard to lung cancer tissue, a substantially increased need for oxygen occurs upon cancer cell proliferation, thus leading to tissue hypoxia which, in turn, stimulates the production of the angiogenic factors that trigger tumor angiogenesis [61,62]. Research outcomes have indicated that Ang-2 concentration is significantly higher in cancer tissues compared to normal lung tissues [23,24,48,50,51,52]. It is significant that a previous meta-analysis that was performed on NSCLC tissues suggested that Ang-2 expression is positively associated with the stage of tumor, tumor differentiation, and lymphatic invasion. Therefore, it was advocated that high Ang-2 levels are significantly associated with the poor prognosis of lung disease [51]. Although the overexpression of Ang-2 in lung cancer tissue is linked with the severity and poor prognosis of lung disease, the increased levels of Ang-2 are highly associated with metastasis, thus facilitating the stratification of patients through the selection of individuals who have a higher risk for metastasis [24,51,63,64]

## 5. Ang-2 and Metastasis

The implication of Ang-2 in lung cancer metastasis was also reflected in in vivo experiments. In particular, the Ang-2 triggers lymph node and lung metastasis in Ang-2 transgenic mice that have been implanted with tumor xerografts. Notably, after the administration of Ang-2-blocking antibodies in the respective mouse models, decreased rates in primary tumor growth, tumor angiogenesis, lymphangiogenesis, and lung metastasis were recorded [65]. One possible explanation for the association of Ang-2 with metastasis is that Ang-2 induces endothelial cell–cell junction disruption, thus facilitating cancer cell spread [65]. A more recent study also revealed that the silencing of Ang-2 transcription with specific shRNA-1 transfection in A549 cell lines eliminates the migration, invasion, and epithelial–mesenchymal transition (EMT) of lung cancer cells [24]. The EMT is a function that augments the metastasis and invasive abilities of cancer cells. During this process, the epithelial cells obtain a mesenchymal phenotype that contains decreased epithelial markers (cytokeratin or E-cadherin), and upregulated mesenchymal indicators (VIM or N-cadherin), or transcription factors (Twist, Snail) [66,67,68]. In particular, after the silencing of Ang-2 in A549 cells, a decrease in epithelial markers, such as E-cadherin, was observed, contrary to the mesenchymal indicators (VIM and N-cadherin) and the transcriptional factors (Twist and Snail), which were found to be upregulated [24]. The impact of Ang-2 on initial tumor development, as well as on the EMT, may render Ang-2 a potential target for future lung cancer treatment. 

## 6. Ang-4 (Ang-3)

Ang-4, and the mouse orthologue, Ang-3, have an agonistic impact on Tie-2, while their effect on angiogenesis and the development of lung cancer is still not well-clarified [30]. However, it has been reported that upregulated Ang-3 inhibits the pulmonary metastasis of Lewis lung carcinoma (LLC), and TA3 mammary carcinoma (TA3) cells, via the inhibition of tumor angiogenesis and the stimulation of tumor cell apoptosis [69]. In addition, a previous study by Olsen et al. [70] revealed that Ang-4 inhibits angiogenesis and constrains the increased interstitial fluid pressure that is generated by bFGF (basic fibroblast growth factor) and VEGF in the human small-cell lung cancer (SCLC) cell line, GLC19.The impact of Ang-4 on lung cancer development is not well-established. However, a previous study concerning the analysis of lung tumor tissue reported that the high tumor cell expression of Ang-4, as well as the high stromal cell expression of Ang-4 and Ang-2, were correlated with improved disease-specific survival, although the biological explanations for these stroma outcomes remain vague [71]. Moreover, it was also recorded that the expression of Ang-2 in tumor cells had no impact on patients urvival [71]. Nowadays, no further analyses have been conducted concerning the concentration of Ang-4 in lung cancer tissues, and no studies have focused on the evaluation of the Ang-4 serum levels in patients diagnosed with lung carcinoma in order to further elucidate the role of the corresponding angiopoietin in lung disease. 

## 7. The Impact of Surgery on Angiopoietins

Clinical trials have demonstrated that surgery is associated with changes in the serum and plasma compositions of patients. Angiopoietins are among the biomolecules affected and, thus, may stimulate tumor recurrences and metastases. In particular, it has been observed that, in NSCLC patients, the postoperative Ang-2 plasma levels are elevated in comparison with the respective preoperative levels. They reach a peak on the 14th postoperative day, and remain elevated for at least eight weeks [72]. The same preoperative and postoperative samples were also used in vivo in human umbilical vein endothelial cells (HUVEC) and human microvascular endothelial cells (HMVEC) in order to evaluate their effect on angiogenesis. The postoperative samples showed a stronger capacity for promoting angiogenesis, tube formation, and proliferation compared to the preoperative samples. Ang-2 plasma blockade ascertained that Ang-2 is responsible for stimulating angiogenesis, while the proliferation progressed independently of Ang-2 [72]. In this study, the type of lung resection has not been taken into account [72]. Kopcczynska et al. [73] also examined the Ang1 and Ang-2 plasma levels before surgical resection, on the 7th and 30th postoperative days. According to their outcomes, the postoperative levels of Ang-2 were significantly higher, while the Ang-1 concentrations did not change significantly. In particular, on postoperative day 7, the Ang-2 levels reached the highest median value, while 30 days after surgical resection, the Ang-2 concentration was decreased, but remained higher in comparison with the baseline median values [73]. 

It is interesting enough to note that the type of lung cancer surgery can affect the angiopoietin concentration as well. Patients with early-stage NSCLC following video-assisted thoracic surgery (VATS) had significantly lower Ang-1levels on the1st and 3rd postoperative days, while the levels in patients who had undergone major lung resection had not been significantly affected. The postoperative levels of Ang-2 in peripheral circulation were higher in patients following open thoracic surgery compared to those following a less invasive type of surgery [74]. Surgery leads to a release in the circulation of angiogenic factors. Though circulating angiopoietins help in wound healing, surgery-induced angiogenesis has a negative impact upon the relapse of the disease. Antiangiogenic administration in a postoperative setting may provide improved clinical outcomes. Further research studies should be done in order to evaluate the possible side effects of antiangiogenic treatment on wound healing. Mathematical models should be used in order to specify the postsurgical day that the angiogenic blockade should begin, and the type of it.

## 8. Discussion

In summary, angiogenesis is involved in a wide variety of neoplasms, since it promotes cancer cell proliferation, resistance to apoptosis, and metastasis. Angiopoietins orchestrate both the normal process of angiogenesis, being responsible for the vascular organization, maturation, and stability, and tumor recurrences and metastases [75,76]. The main cause of death in patients with stage I NSCLC is the angiogenesis-induced conversion of micrometastases (<1 mm) into macrometastases (1 mm) [77]. 

The impact of angiopoietins on the development of lung cancer has gained increased popularity. Differences in the serum or plasma levels of angiopoietins have been detected in patients diagnosed with NSCLC compared to controls. Although little is known concerning the association of Ang-1 and Ang-4 in lung disease, the vast majority of research studies have focused on the association of Ang-2 with NSCLC, since elevated levels of the respective angiopoietin are detected in NSCLC cases, either in the serum or tissue of the examined patients [23,24,25,51,52,53,54]. It has been concluded that Ang-2 plays a fundamental role in primary tumor growth, tumor angiogenesis, and lymphangiogenesis, while higher levels of Ang-2 are related to the advanced stages of NSCLC, and poor prognosis as well [51]. Additionally, high levels of Ang-2 sufficiently indicate the presence of metastasis. Taking all these data into consideration, it can be stated that Ang-2 is a marker for NSCLC development, progression, and cancer spread. It is important to highlight that silencing Ang-2 migration and invasion and attenuating the epithelial–mesenchymal transition of the lung cancer cells renders Ang-2 an appropriate future target for lung cancer therapy.

NSCLC patients treated with anti-VEGF agents, both as a first-line and as a second-line therapy, have showed response. Recent studies provide accumulative evidence that ANG-2 may be crucial in acting synergistically with current antiangiogenic treatments for various solid tumors [78,79,80]. Anti-ANG-2 therapy, in combination with monoclonal antibodies simultaneously targeting other angiogenic factors, including VEGF, TIE2, or ANG-1, may enhance the effect on lung cancer therapy as well. Immunotherapy, mainlythe immune checkpoint ligand, programmed celldeath ligand1 (PD-L1), dramatically changed lung cancer treatment. In vivo experiments have indicated that the dual administration of ANG-2 and VEGF inhibitor stimulated the response to immunotherapy by promoting antitumor immunity and by sensitizing mouse tumors to PD-L1 [81].

Both immunotherapy and antiangiogenic treatments have serious side effects and, unfortunately, not all patients benefit from their administration [82]. Moreover, resistance to antiangiogenic therapy is a common problem. Great research efforts have been made in order to discover biomarkers to predict patient responses, and as a strategy to circumvent resistance to angiogenic inhibitors. Angiopoietins may prove beneficial as therapeutic targets, while they may also serve as markers of the response to targeted complementary therapy. Interestingly, Ang-2 is affected by the type of performed surgery, since higher levels of the angiopoietin are detected in more invasive surgical procedures. As a result, we propose that the Ang-2 levels followed in lung cancer incidences, as well as in surgery, should be considered together in order to further improve the prognosis and survival of patients.

In conclusion, in the present review, we provide important insights regarding the role of angiopoietins as biomarkers for NSCLC development. It is anticipated that the analysis of angiopoietin levels, and particularly that of Ang-2, will be essential in each individual patient in order to collect crucial information concerning lung cancer progression, metastasis, and treatment.

## Data Availability

Not applicable.
